# Prenatal diagnosis after high chance non-invasive prenatal testing for trisomies 21, 18 and 13, chorionic villus sampling or amniocentesis? – Experience at a district general hospital in the United Kingdom

**DOI:** 10.1016/j.eurox.2023.100211

**Published:** 2023-07-01

**Authors:** Collins Ejakhianghe Maximilian Okoror, Suruchi Arora

**Affiliations:** Department of Obstetrics and Gynaecology, Royal Berkshire Hospital, Craven road, Reading RG1 5AN, UK

**Keywords:** Non-invasive prenatal testing (NIPT), Antenatal screening/diagnosis, Chorionic villus sampling (CVS), Amniocentesis, Invasive prenatal testing/diagnosis, Confined placental mosaicism (CPM)

## Abstract

The non-invasive prenatal testing (NIPT) analyses cell-free DNA (cfDNA) derived from the placental tissue in the maternal circulation. Though highly sensitive and specific, a major limitation is in cases of confined placental mosaicism (CPM). Whether to perform chorionic villus sampling (CVS) or amniocentesis to confirm a positive NIPT result is controversial. One major drawback of CVS is that cytogenetic diagnosis may not always reflect the true chromosomal make-up of the fetus. This work, therefore, proposes the use of amniocentesis in the presence of normal ultrasound findings, and the option of either CVS or amniocentesis when there are abnormal USS findings.

## Highlights

• Both NIPT and CVS asses the placenta as a surrogate for fetal status.
• The placental genetic makeup is not always same as that of the fetus, a condition that can result in false positive result.
• Utilizing this proposed framework will reduce the risk of false positive result as well as improve quality of care following high chance NIPT.

## 1 Background

Prenatal screening and diagnosis involve testing for conditions in a fetus before it is born to detect causes of birth defects, abnormalities, and genetic conditions. Testing procedures can include non-invasive techniques, such as ultrasound scan, maternal serum testing like the first trimester combined screening test (CST) and the second-trimester quadruple screening test (QST), and non-invasive prenatal testing (NIPT); or the invasive techniques, such as chorionic villus sampling (CVS) and amniocentesis. Invasive techniques generally carry risks, including the rare instances of fetal loss which is estimated at up to < 0.5 % [1], [2], [3]. The non-invasive techniques, with no risk to fetus and pregnancy, are often first employed to screen while those with high chance results can then proceed to have the invasive testing if they desire.

The NIPT analyses cell-free DNA (cfDNA) derived from the placental tissue in the maternal circulation. cfDNA is the DNA that exists in the plasma or other body fluids as short fragments. It is different from the DNA which is enclosed within the nucleus of an intact cell. cfDNA fragments are released during a range of cellular processes, including apoptosis, necrosis and microparticle secretion from all organs [4]. The maternal plasma cfDNA which is known as the total cell-free DNA (cfDNA), contains the combination of maternal and fetal sources of cfDNA. The source of fetal DNA is the trophoblast [5], [6], [7], [8], while the hematopoietic system constitutes the major source of maternal DNA [9]. All maternal organs contribute some cfDNA into maternal plasma, including solid tumors [10]. In most cases, the placental DNA will be the same as the baby’s DNA and this fetal contribution is known as the cell-free fetal DNA (cffDNA). The cffDNA can be identified in the maternal blood as early as 5 weeks of gestation and clears within a few hours after the pregnancy, making it appropriate for pregnancy-specific testing. This comprises only about 10–15 % of the cfDNA. The test poses no risk to the baby as the only blood drawn comes from the pregnant woman rather than the fetus. It can be used to screen for Trisomy 13, 18 and 21 as well as sex chromosome aneuploidy and single-gene disorders. While NIPT is now offered as part of the routine first-line screening measures for pregnant women in some countries including the United States (US) [11], in others including the United Kingdom, it is currently offered to women only following a higher chance result from CST or QST for T21, 18 and 13 [12], [13].

## 2 Non-invasive prenatal testing (NIPT) versus combined screening test (CST)

The first trimester combined screening test (CST) is still mainly used as the first-line screening method in a lot of countries including the UK. This is carried out between 11 + 0 weeks and 13 + 6 weeks gestation or when the fetal crown-rump length (CRL) measures between 45 mm and 84 mm. The risk result is determined based on pre-test odds of maternal age and previously affected pregnancy combined with ultrasound measurement of the nuchal translucency (NT), and maternal serum analytes (free beta-human chorionic gonadotropin [fb-hCG], and pregnancy-associated plasma protein A [PAPP-A]). The chance is considered high if between 1 in 2 and 1 in 150. Women with higher chance results are given the options of no further testing, having further screening testing with NIPT or invasive diagnostic testing. The accuracy of the combined test has been quoted as 90 %, 97 % and 92 % for trisomies 21, 18 and 13 respectively, at a false positive rate (FPR) of 4–5 % [14], [15]. On the other hand, systematic reviews and meta-analysis on NIPT have reported higher sensitivities of more than 99 % for trisomy 21 and more than 97 % for trisomies 18 and 13, with specificities > 99 % for the three trisomies in singleton pregnancies, and slightly lower sensitivities for twin pregnancies [16], [17], [18], [19]. NIPT, therefore, serves as a reasonable measure to further triage women following high chance CST to avoid unnecessary exposure of all these women to the higher risk of invasive testing [20]. Some countries, including the United States, offer the option of the NIPT both as a first-line screening for all pregnant women in their first trimester or as the second line for those with high chance CST [11]. Though, high sensitivity and specificity have been quoted for NIPT, this does not hold in the general obstetric population where there is a lower prevalence of trisomy. The positive predictive value (PPV) is much lower among this population. This stresses the fact that NIPT is only a screening tool and requires confirmation of positive results with invasive prenatal diagnosis before major decisions such as discontinuing the pregnancy are made.

## 3 Limitations of NIPT

### 3.1 False negative results

NIPT can be influenced by some factors which cause an actual or relative decrease in the fetal fraction of the cfDNA, resulting in false-negative NIPT results. The measurement of the fetal fraction is vital to sample quality control and statistical confidence. In order to produce a meaningful result, it is important to ensure that placental cfDNA is measurable in the maternal plasma in adequate quantity and this can be done by measuring the fetal fraction. The fetal fraction is influenced significantly by maternal weight. Maternal weight has a proportional association with the fetal fraction. As maternal weight increases, there is proportional decrease in the fetal fraction of DNA and consequently, the likelihood of false-negative results [21], [22]. It has also been reported that the fetal fraction decreases with increasing parity, maternal age, vitamin B12 deficiency and active autoimmune disease such as systemic lupus erythematosus (SLE) [7], [23], [24], [25], [26], [27]. Consequently, NIPT is contraindicated in pregnant women who have cancer (unless in remission), received a blood transfusion in the previous 4 months, had a bone marrow or organ transplant, immunotherapy in the current pregnancy (excluding intravenous immunoglobulin (IVIg) treatment), had donor stem cell therapy, or a vanished twin pregnancy. Also, NIPT should not be carried out in pregnant women with Down’s syndrome or a balanced translocation or mosaicism of T21, T18 or T13 which could potentially be problematic as these may not be physically obvious without a prior genetic diagnosis. Fetal mosaicism and triploidy are also associated with false negative results [28], [29]. A case was reported of a discrepancy between the NIPT result and CST. The NIPT was reported as low risk for trisomy 13, 18 and 21 while the CST demonstrated a high risk for trisomy 21. An amniocentesis done showed trisomy 21 in the quantitative fluorescence-polymerase chain reaction (QF-PCR). The patient had a medical termination of the pregnancy (MTOP) and the placenta microarray revealed the presence of mosaicism of trisomic 21 (67 % of the cells) [30]. NIPT distinguishes pregnancies affected by aneuploidies from those that are not through quantitative differences in the number of DNA fragments from different chromosomes. For example, fetuses with trisomy 21 will have a quantifiable and statistically significant increase in the number of chromosome 21 DNA fragments. The numerical differences in the number of DNA fragments could be low in mosaicism resulting in a false-negative result. To reduce the occurrence of a false negative result, a minimum threshold of fetal fraction is employed in interpreting NIPT results. This threshold varies by assay but is typically between 2 % and 4 %. With a higher fetal fraction, there is a greater statistical separation of aneuploid and euploid pregnancies, providing greater confidence in the result. As the fetal fraction is low at early gestation, this must be taken into consideration when requesting or counseling a patient for NIPT. Typically, between 10 and 20 weeks of gestation, the average fetal fraction is 10–15 % [21], [31]. Hence, the practice is, in some places, to avoid NIPT before 10 weeks of gestation.

### 3.2 False positive result

One of the major potential limitations of the NIPT is in cases of confined placental mosaicism (CPM). This is a situation where there is an inconsistency between the chromosomal makeup of the cells in the placenta and that in the fetus. This can occur through a mitotic nondisjunction event or aneuploidy rescue. Though NIPT has high sensitivity and specificity, false-positive results can occur in cases of CPM. CPM may also be a marker for uniparental disomy. Wu et al. [32] studied placental specimens sampled from eight pregnancies with confirmed false-positive NIPT results using copy number variation sequencing (CNV-seq) and single nucleotide polymorphism array (SNP-array). Five of them were proven to be CPM involving trisomy 9, 13, 21, 22 and X. Malvestiti et al. [33], evaluating 60347 chorionic villi sampled over a 14-year period, reported 1317 mosaic cases. Of these, 1001 were subsequently investigated with amniocentesis. They reported CPM in 870 of them and only 33 % of them were true fetal mosaicism. In another study looking at the performance of NIPT following a high chance result for trisomy 21 from combined first trimester screening, a 100 % detection rate was reported with a false positive rate of 5.6 % which were possibly CPM [34]. The Royal College of Obstetricians and Gynecologists (RCOG) quotes an overall incidence of confined placental mosaicism as 1–2 % [1].

## 4 Prenatal diagnosis after high chance NIPT for trisomies 21, 18 and 13 – chorionic villus sampling (CVS) or amniocentesis?

Given that NIPT can result in false positives, positive results should be confirmed with invasive testing before any irreversible procedure is performed [20]. It also further stresses the fact that NIPT is a screening test and extensive counseling about this, and the possibilities of false-negative and false-positive results is important for a patient to make an informed decision before and after the result, and guide against medico-legal issues if there is a discrepancy at birth.

The difficulty then arises in deciding which of the diagnostic tests to conduct following a high chance NIPT result for trisomies 21, 18 and 13. Whether to perform CVS or amniocentesis to confirm a positive NIPT result is controversial. Current guidelines and recommendations have not specified either, but suggestions seem to be based on the gestational age at which the patient presents. Current advice is that such women should be offered the options of prenatal diagnosis (PND) or no further testing following a higher chance or “No result” NIPT results [1], [2].

CVS and amniocentesis have been well recognized as procedures for definitive diagnosis in patients with a high-risk result following NIPT. Both procedures have been reported to be safe options when there is higher chance NIPT result with a miscarriage rate of < 0.5 % [1], [3]. CVS, also known as placental biopsy, involves taking some placental tissue for cytogenetic diagnosis. The practice, until now, is that non-mosaic chromosomal abnormality from CVS should be reported as positive for the abnormality to facilitate the patient’s choice of whether to continue with or end the pregnancy. The major drawback of this practice is that cytogenetic diagnosis from CVS may not always reflect the true chromosomal make-up of the fetus due to confined placental mosaicism. There have been reports of cases where CVS reported non-mosaic trisomy 21, while amniocentesis reported normal karyotype and the baby was normal after delivery [35]. Some authorities, including the Royal College of Obstetricians and Gynaecologists (RCOG) and the Public Health England (PHE), have advised that a full karyotype or result of cell culture be awaited before making any decision if there are no structural anomalies, even when QF-PCR results following CVS suggest a chromosomal anomaly [1], [12]. The result of QF-PCR following CVS is usually available within 3 days. The inability to make decision based on it when it reports an abnormality on a background of a normal detail scan, places a limitation in its utilization. The result of full karyotype or cells culture can take two to three weeks or more, considerably prolonging the waiting time and period of intense anxiety and psychological distress for the couples and their families. In addition, this is at the expense of high costs, of both human and material resources. Another practice employed by some is to proceed with CVS following high chance NIPT and to examine all cell lines of both an uncultured sample, using fluorescence in situ hybridization (FISH) and short-term culture as well as a long-term culture of the sample. If the results all show aneuploidy, the results are reported to the patient. Otherwise, if the results are also mosaic, amniocentesis is recommended and analyzed by both FISH and karyotype [36]. Apart from this approach not completely ruling out discrepancies between the placental and fetal genotype, it also has the same potential implication highlighted earlier of prolonged waiting time, delayed decision making and a huge cost. Amniocentesis, on the other hand, analyses the fetal DNA from the amniotic fluid and is, therefore, devoid of the false positive result from placental mosaicism. Levett et al. [37] compared amniocentesis QF-PCR results to karyotype in over 5000 patients. They reported that QF-PCR from amniocentesis correctly identified 100 % of cases of trisomies 21, 18 and 13. Amniocentesis has been well recognized as a better reflection of the fetal DNA and several bodies have recommended it as an option when CVS result suspected any of T21, T18 or T13 with no unexpected findings on ultrasound scan consistent with any of these conditions. Amniocentesis can be offered only after 15 weeks, which may involve a significant wait after receiving a high risk NIPT result, but PCR result being 100 % reliable enables the decision making to be available to couple faster than if they potentially had a CVS at 13 weeks, followed by a 3 week wait for the karyotype result.

## 5 Experience at a district general hospital in the UK

The NHS UK currently does not routinely offer NIPT as a first-line screening modality for pregnant women [12]. Women are offered first trimester combined screening, while those that register late or could not have the nuchal translucency of their babies accurately measured are offered 2nd trimester quadruple screening. Those with a high chance CST or QST are offered the options of further screening with NIPT, invasive testing with either CVS or amniocentesis, or no further testing. Those with diagnosed anomalies are followed up by the fetal medicine team till the end of the pregnancy and subsequent pregnancies as appropriate in those with higher chance of recurrence in which prenatal diagnosis can be offered. This has been the practice at the Royal Berkshire Hospital (RBH), Reading, a district general hospital in South-East England, delivering high quality acute medical and surgical services to over 500,000 patients across West Berkshire, an area extending from Newbury in the West to Henley-on-Thames in the East, and including Wokingham and parts of Hampshire to the South and parts of Oxfordshire to the North. The Trust is one of the largest general hospital Trusts in the country. The Obstetrics and Gynecology department, in addition to other specialized services, offers fetal medicine care.

**Fig. 1:**
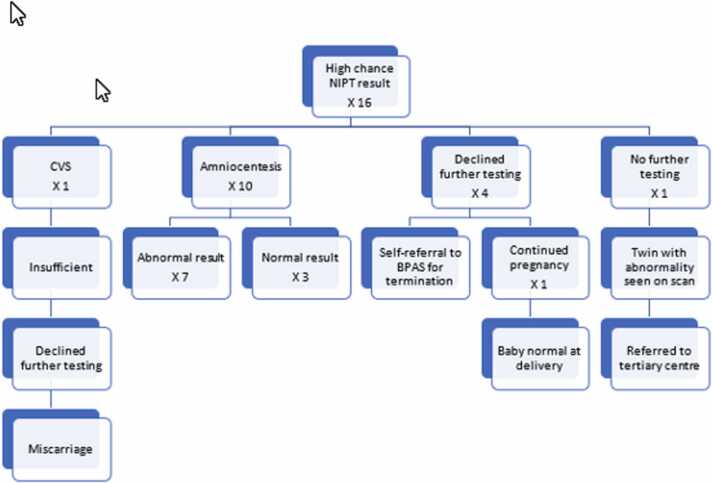
Outcome of high chance NIPT result at the Royal Berkshire Hospital.

Recently, there has been an increasing number of women presenting with primary NIPT results done privately. Consequently, the number of high chance results from NIPT has also been on the increase. To address the problem of a better diagnostic test to offer this cohort of women, we did a retrospective search of outcomes in patients at the Royal Berkshire Hospital with a high chance NIPT result for trisomies 21, 18 and 13 between June 2021 and May 2022. Findings are shown in Fig. 1. There were 16 cases of high risk NIPT results during this period. Out of these 16 high risk NIPT results at RBH, 4 babies were identified to have normal chromosomes, 3 by amniocentesis and 1 by postnatal testing. Of these 16 NIPT positive cases, 14 of them had positive combined screening test, one a positive quadruple test and the last didn’t have serum screening. Two of them had abnormal ultrasound scan findings. QF-PCR and karyotyping were done following amniocentesis and the results of both testing were in agreement. Microarray of the fetal tissue was carried out following miscarriage of the patient with insufficient sample from CVS and this confirmed trisomy 18. Overall, the false positive rate of NIPT in our center during this one-year period was identified to be 25 % (4/16). It is this high number which has alerted us to changing the way we counsel our couples after high risk NIPT results and compelled us to share our experience with the wider Fetal Medicine community.

## 6 What is the way forward?

**Fig. 2:**
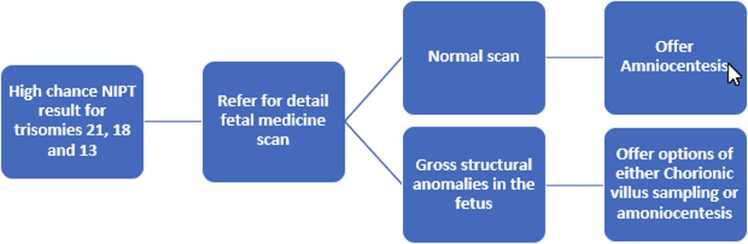
Proposed path for confirmation of high chance NIPT result for trisomies 21,18 and 13.

From the foregoing, it suffices to say that while CVS can be performed earlier than amniocentesis, we risk the error of NIPT when CVS is done following high chance NIPT since both assess the fetal placenta rather than the fetus directly. We, therefore, recommend a detailed scan by a fetal medicine specialist following a high chance NIPT result for trisomies 21, 18 and 13 (Fig. 2). Where there are gross structural anomalies in the fetus, the option of either CVS or amniocentesis should be explored based on the gestational age at presentation and preference of the woman after informed counseling. However, where a detailed scan does not show any obvious fetal anomaly, we recommend offering amniocentesis as the preferred method of choice due to its several advantages, such as direct analysis of the fetal DNA rather than the placenta and thus avoiding the risk of discrepancies caused by CPM, and the availability of results within 2–3 days of testing instead of waiting for two to three weeks for CVS culture.

Counseling couples with higher chance results is very important both in guiding them to make an informed decision as well as providing some emotional support. Couples who opt to have NIPT should be aware that it is a screening test which means that further testing will be required to confirm any higher chance results [2]. The limitation of this test should be appropriately explored with the couple to guide their decision. They should be aware that the high sensitivity and specificity of the test means that it can serve as a good screening tool to reduce the need for invasive testing and its potential complications. Typically, a couple’s initial information on the options of prenatal genetic screening and diagnosis is by their obstetrician or midwife [38], [39]. Consequently, they continue to be the first point of contact for information about new testing methods. A number of professional guidelines have said that women who are offered prenatal screening and testing for genetic disorders should be educated and counseled before testing so that they are aware of the benefits, risks, and limitations of the various testing options [1], [2], [40], [41], [42], [43], [44]. After that, they should make decisions based on their own beliefs and choices. Education and counseling for genetic testing ought to be provided promptly following a higher chance NIPT result in order to make optimal use of the time available to weigh testing decisions and provide appropriate follow-up. The PHE suggests that the appointment be completed by a healthcare professional familiar with NIPT and the screening pathway within three working days [2]. Generally, pre-test counseling should get couples ready for a potential report of an abnormality. Care providers should be ready to give these results, offer post-test counseling, and make referrals.

Women typically choose NIPT to screen for Down syndrome since they are unfamiliar with other disorders [45]. Genetic counseling before prenatal diagnosis by amniocentesis and CVS has always had as one of its primary objectives preparing couples for potential unexpected outcomes, which can be a factor in practically any genetic testing circumstance. Providers should be prepared to give couples time to react and comprehend the significance of the results as reports of abnormality have been linked to significant anxiety. They should be made aware of the possibility of diagnostic testing for the purpose of confirming results, and genetic counseling made available. The care provider should at the very least provide some information when discussing the alternatives for prenatal invasive testing during the pretesting counseling. This should include the fact that NIPT is a screening test, thus more testing is necessary to confirm a diagnosis before making an irreversible choice; genetic testing is discretionary, the choice to undertake or avoid it should be based on the individual patient's beliefs and desires; the significance of a report of abnormality and options of follow-up; limitations and risks of the various diagnostic testing; accessibility to genetic counseling to offer more information and risk evaluation to help with making a decision and follow-up plan; and the possibility of detecting other genetic problems not detectable by NIPT [1], [2], [20], [46].

PND testing provides near-conclusive results for those with a higher chance NIPT results. However, patients should be made aware that CPM may occur following CVS. Consequently, abnormal cells that do not correspond to the fetal karyotype may be found in the placenta in these instances. Additionally, it has been demonstrated that the cytotrophoblast is the source of cell-free DNA in maternal blood, making it "placental" rather than fetal. In some instances, placental mosaicism can complicate NIPT screening [47].

This counseling should be provided by the fetal medicine (FM) team, which should include the FM specialist and the FM specialist screening midwives. In a model, the FM specialist screening midwives are the couple's first point of contact after a higher chance result. They discuss the result and offer options going forward, including providing additional sources of information. After that, the woman is referred to the FM specialist for additional counseling, scans, and PND, if the couple so chooses. With this strategy, there is timely contact with the couple following the result and allows them time to consider their options while they wait to talk to the FM specialist. There is evidence to suggest that nurses and midwives were more likely than obstetricians and geneticists to provide non-directive advice [48]. The couple will have a greater opportunity to have all of their options presented to them fairly equally during their first contact with the FM screening midwives to help them make an informed decision. The psychological component following a diagnosis of an abnormality cannot be overemphasized as it underpins the ability of the couple to make decisions in index pregnancy as well as prepare them for future pregnancies. During the time between diagnosis and decision, the couple is able to receive timely emotional support by making initial contact with the screening midwives.

Post-testing counseling and who should provide this following PND should depend on the result. Those with normal results can be contacted by the FM screening midwives and reassured. In situations where there have been abnormalities detected, discussion with the fetal medicine specialist and geneticist will be strongly recommended to help understand what this means for them and if further testing is advised. The report of an abnormal result should trigger communication and support of the fetal medicine team. This is important to ensure that the couple is given appropriate and adequate information to enable them to make decisions as well as provide necessary emotional support throughout the pregnancy. The patient should be contacted by the geneticist who will counsel her on the diagnosis, and outlook of the condition including prognosis and risks of recurrence. The geneticist is therefore able to discuss parental testing where appropriate and offer advice for future pregnancies. As this could be emotionally draining, it is important that the couple is supported throughout the period regardless of the decision they make. Fetal medicine screening midwives can offer these services and where it is deemed important, the services of a clinical psychologist can be employed. Advances in the field of fetal medicine have resulted in increasing demand for the services and hence a high patient burden. Initial contact with the screening midwives will ensure that the delays are removed or kept at the barest minimum and that a timely appointment is made for the couple to be seen at the fetal medicine clinic where further discussion can take place. The discussion at this clinic should at the minimum include details of the diagnosis, prognosis of the condition, options for management of index pregnancy, and future pregnancies. This may sometimes involve discussion with the couple in a setting involving other specialties, especially where the couple opts to continue the pregnancy. This will help the couple to be involved in the care of the baby and prepare for appropriate treatment that may become necessary during pregnancy and post-delivery. Management of the index pregnancy can sometimes include a transfer to another center where the baby can receive adequate care immediately post-delivery.

## 7 Conclusion

CVS is often done following a high chance NIPT result for trisomies 21, 18 and 13 due to earlier gestation for procedure. One major drawback of this is that cytogenetic diagnosis from CVS may not always reflect the true chromosomal make-up of the fetus. Amniocentesis has been reported as the true reflection of the fetal DNA and have been recommended as an option when CVS result suspected aneuploidy with no unexpected findings on ultrasound scan consistent with the conditions. We are, therefore, proposing a referral to a fetal medicine specialist for detail fetal scan following a high chance NIPT result for trisomies 21, 18 and 13. Where there are findings of fetal abnormalities consistent with the condition, the options of CVS and amniocentesis should be offered to the patient based on gestational age and her preference. On the other hand, amniocentesis should be the preferred procedure in the presence of normal ultrasound findings.

## Funding

The authors received no fund for this work.

## Declaration of Competing Interest

The authors hereby declared that we have no conflict of interest in undertaking this work and that no funding was received for this work.
